# Characterization of zinc amino acid complexes for zinc delivery in vitro using Caco-2 cells and enterocytes from hiPSC

**DOI:** 10.1007/s10534-017-0033-y

**Published:** 2017-07-17

**Authors:** Ann Katrin Sauer, Stefanie Pfaender, Simone Hagmeyer, Laura Tarana, Ann-Kathrin Mattes, Franziska Briel, Sébastien Küry, Tobias M. Boeckers, Andreas M. Grabrucker

**Affiliations:** 1 WG Cellular Neurobiology and Neuro-Nanotechnology, Department of Biological Sciences, University of Limerick, Limerick, Ireland; 2 Bernal Institute, University of Limerick, Limerick, Ireland; 3 Institute for Anatomy and Cell Biology, Ulm University, Ulm, Germany; 4 CHU Nantes, Service de Génétique Médicale, Nantes, France

**Keywords:** Zip4, Acrodermatitis enteropathica, Absorption, Gastro-intestinal, Enterocyte, hIPSC

## Abstract

Zn is essential for growth and development. The bioavailability of Zn is affected by several factors such as other food components. It is therefore of interest, to understand uptake mechanisms of Zn delivering compounds to identify ways to bypass the inhibitory effects of these factors. Here, we studied the effect of Zn amino acid conjugates (ZnAAs) on the bioavailabilty of Zn. We used Caco-2 cells and enterocytes differentiated from human induced pluripotent stem cells from a control and *Acrodermatitis enteropathica* (*AE*) patient, and performed fluorescence based assays, protein biochemistry and atomic absorption spectrometry to characterize cellular uptake and absorption of ZnAAs. The results show that ZnAAs are taken up by AA transporters, leading to an intracellular enrichment of Zn mostly uninhibited by Zn uptake antagonists. Enterocytes from *AE* patients were unable to gain significant Zn through exposure to ZnCl_2_ but did not show differences with respect to ZnAAs. We conclude that ZnAAs may possess an advantage over classical Zn supplements such as Zn salts, as they may be able to increase bioavailability of Zn, and may be more efficient in patients with *AE*.

**Electronic supplementary material:**

The online version of this article (doi:10.1007/s10534-017-0033-y) contains supplementary material, which is available to authorized users.

## Introduction

 Research from the last decades clearly shows that Zn has a vital role in neonatal and postnatal development, and adult life. Zn is an essential trace element in humans and animals, and is involved in countless metabolic and signaling pathways within the body. However, a particular role of Zn in the immune system and brain has been reported (Prasad 2014).

Two major pools of Zn can be found within the body. One pool that contains about 90% of the body’s Zn is exchanging Zn slowly with the blood. The second pool that rapidly exchanges Zn with the plasma contains the other 10% of Zn and is the one that is especially reactive to the amount of absorbed Zn from the diet (Hambidge et al. 1986). This Zn supply is dependent on the amount and bioavailability of Zn. In a western mixed diet consisting of commonly consumed foods, the bioavailability of Zn is about 20–30% (Gallaher et al. 1998). Various compounds and substances present in the diet can inhibit Zn absorption. For example, phytates such as inositol hexaphosphates and pentaphosphates bind to Zn and reduce its availability (Lönnerdal 2000; King 2007). Further, high fructose corn syrup (HFCS) is able to influence Zn absorption, as does Cu, which has an antagonistic relationship with Zn (Hill and Matrone 1970; Mills 1985; Hall et al. 1979; Huster 2010). Moreover, Ca and Fe might interfere with the absorption of Zn (Wood and Zheng 1997; Whiting and Wood 1997; Argiratos and Samman 1994; O’Brien et al. 2000; Fischer Walker et al. 2005; Hambidge et al. 1983). Additionally, folic acid, a nutrient commonly prescribed and supplied at higher levels during pregnancy has been shown to decrease Zn absorption (Ghishan et al. 1986; Simmer et al. 1987; Krebs 2000).

Regarding Zn in supplements, the bioavailability can vary from very low (e.g. zinc oxide) to comparatively high (e.g. zinc salts such as zinc acetate) and is influenced by multiple factors (competition with other minerals as well as Zn itself supplied through dietary sources, inhibition by food substances) (Lönnerdal et al. 1984). An ideal Zn supplier should therefore use other routes as those potentially inhibited and saturated by Zn and other competitive trace metals, such as classical Zn transporters.

Here, we investigated uptake and absorption of three compounds with Zn bound to amino acids (ZnAAs): Zn-glutamate (ZnGlu), Zn-lysine (ZnLys) and Zn-methionine (ZnMet). ZnAAs have been studied in the past. For example, it was shown that 100 ppm Zn from ZnAAs additional to 100 ppm Zn from ZnSO_4_ in sow diets may increase pigs born and weaned per litter (Payne et al. 2006), and that ZnMet supplemented diet slightly improves intestinal morphology of weaned pigs (Metzler-Zebeli et al. 2010). Further, ZnAAs showed positive effects regarding production parameters in dairy cows (Osorio et al. 2016; Nayeri et al. 2014), and a higher bioavailability of Zn from ZnAA compared to Zn from ZnSO_4_ was shown in broilers (Star et al. 2012). However, the different possible routes of uptake of these ZnAAs have not been investigated in detail so far.

Under physiological conditions, Zn is taken up in the gastrointestinal (GI) tract into enterocytes by Zn transporters of the Zrt- and Irt-like protein (ZIP/SLC39A) family that mediate the uptake, while Zn transporters of the Zinc Transporter (ZnT/SLC30A) family mostly mediate removal. For example, the ZIP4 (SLC39A4) transporter plays a major role in Zn absorption in the small intestine (Cousins and McMahon 2000). Mutations in ZIP4 may cause *Acrodermatitis enteropathica* (*AE*), a rare and, if left untreated, lethal autosomal recessive genetic disorder of Zn uptake (Andrews 2008). The release of Zn from enterocytes into the blood stream, in turn, is mediated by ZnT1 (SLC30A1) (Cousins 2010).

Amino acids, for example from digested proteins, are taken up by at least four sodium-dependent amino acid transporters, and sodium-independent transporters, mediating the uptake of acidic, basic, neutral amino acids. We investigate Zn bound to glutamate (Glu), lysine (Lys) and methionine (Met). Glu is a non-essential AA and classified as acidic AA. l-Lysine (α,ε-diaminocarpoic acid) and l-Methionine (2-amino-4-(methylthio)butanoic acid) are essential AAs. Lys is a basic AA, whereas Met belongs to the hydrophobic AAs.

As in vitro model for Zn uptake and absorption, we use the human intestinal cell line Caco-2. Caco-2 cells have been used to assess Zn metabolism previously (Zödl et al. 2003) and are recognized by the FDA as a validated model to study drug absorption in humans (Corti et al. 2006). In addition, we use human enterocytes differentiated from a healthy individual and a patient with *AE.* For in vivo studies, we use mouse models that were fed different diets containing antagonists with and without ZnAAs for 8 weeks. The Zn transporters in the intestines of mice and humans are highly conserved not only in their sequence but also the different isoforms. We hypothesized that Zn linked to AAs might be taken up by AA transporters to some extent and thus may ameliorate inhibition of Zn absorption in the presence of antagonists.

## Results

### Zn deficiency can be induced by uptake antagonists in vivo

Rather than the total concentration, the bioavailability of Zn in the diet plays a major role for the Zn status of the body. As a proof of principle, we fed female wild type C57BL/6 mice 3 different diets for 9 weeks. The diet was started in 10 weeks old animals. Diet 1 (Control) was a standard laboratory diet containing all necessary vitamins and minerals including 41 mg/kg Zn, 0.72% Ca, 113 mg/kg Fe, 4.5 mg/kg phytates, and 0.7 mg/kg folic acid. Diet 2 (Zn deficient) was the same standard laboratory diet except Zn was reduced to 19 mg/kg. Diet 3 (Zn inhibitor) was a standard laboratory diet containing the 41 mg/kg Zn, but with increased levels of Zn uptake antagonists (1.13% Ca, 503 mg/kg Fe, 9.5 mg/kg phytates, and 1.9 mg/kg folic acid). A complete list of ingredients can be found as supplementary data (Supplementary Data 1).

**Fig. 1:**
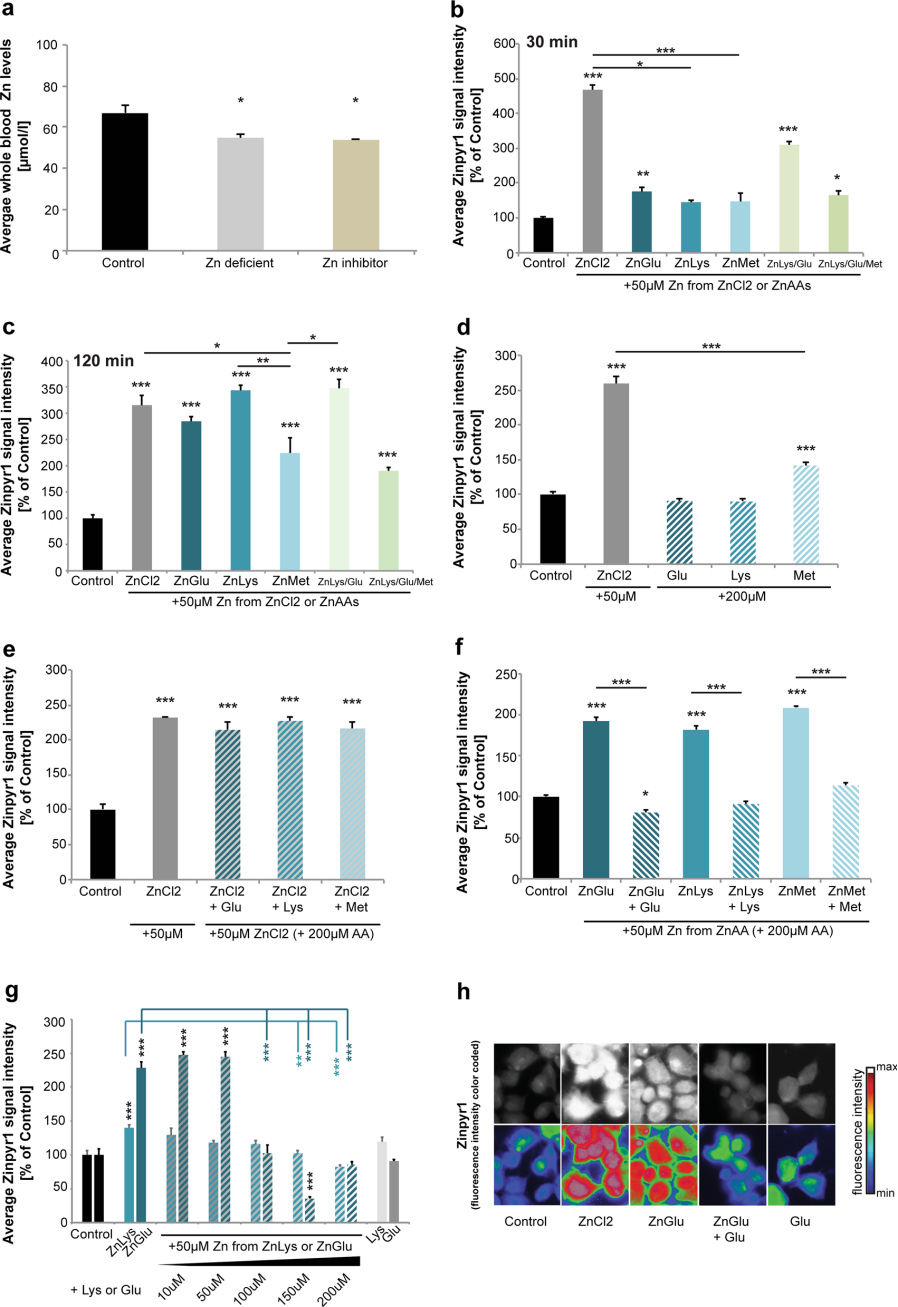
Uptake of Zn from ZnAAs in Caco-2 cells. **a** Blood levels of Zn from mice on different diets for 9 weeks. Whole-blood Zn levels were investigated by AAS in three animals per group. Animals on a Zn deficient diet (Diet 2) show significantly reduced Zn levels compared to mice on the control diet (Diet 1). Mice on the control diet with increased levels of Zn uptake antagonists (Diet 3) similarly show a significant reduction in blood-zinc levels. **b**–**h** Zinpyr-1 fluorescence intensity of Caco-2 cells incubated for 30 min with ZnCl_2_ solution (50 μM) or ZnAAs delivering an equivalent of 50 μM Zn^2+^. **b** ZnCl_2_ solution, ZnGlu, and ZnLys/Glu and ZnLys/Glu/Met significantly increase intracellular Zn. A trend for an increase was seen after application of ZnLys and ZnMet. ZnCl_2_ solution increases intracellular Zn levels significantly more compared to ZnLys and ZnMet (*n* = 10 cells per group). **c** After 120 min, ZnCl_2_ solution and all ZnAAs lead to a significant increase in intracellular Zn (*n* = 10 cells per group). **d** Application of Glu and Lys alone do not lead to differences in Zn compared to untreated controls. The application of Met alone results in a significant increase in intracellular Zn compared to controls, but significantly less than seen with ZnCl_2_ solution (*n* = 10). **e** The significant increase in intracellular Zn levels provided by ZnCl_2_ solution is not affected by the presence of a surplus of AAs in the medium. **f** A significant increase in intracellular Zn after application of ZnGlu, ZnLys, and ZnMet was not present when ZnGlu, ZnLys, and ZnMet were applied together with Glu, Lys and Met, respectively. **g** The increase in intracellular Zn is reduced in a concentration dependent manner by co-application of 10, 50, 100, 150 and 200 μM Lys or Glu, respectively (*n* = 10). **h** Exemplary images for Caco-2 cells stained with Zinpyr-1 are shown. The *bottom row* shows the Zinypr1 signal intensity color-coded

Whole-blood Zn levels were investigated by AAS in three animals per group (Fig. 1a). The results show that animals on a Zn deficient diet (Diet 2) had significantly reduced Zn levels compared to mice on the control diet (Diet 1) (one-way ANOVA, F = 8.740, *p* = 0.017, Post hoc analysis: Control vs. Zinc deficient, *p* = 0.0461). Interestingly, mice on the control diet that contained increased levels of Zn uptake antagonists (Diet 3) showed a reduction in blood-zinc levels similar to the mice on the Zn deficient diet (Control vs. Zinc inhibitor, *p* = 0.0307). Thus, despite adequate supply of Zn in the diet, the presence of increased levels of Zn uptake antagonists was sufficient to induce Zn deficiency in mice. To overcome the antagonistic effects and thus to increase bioavailability of Zn, delivery of Zn using ZnAAs may be a promising approach.

### Zn from ZnAAs is taken up by AA transporters in Caco-2 cells

In the ZnAAs used in this study, the complex with Zn is formed between the amino acid group and the alpha nitrogen. Thus, although the side group may increase the stability, it is not needed for binding. In a first set of experiments, we investigated the possibility to visualize ZnAA complexes in cell free conditions by fluorescent probes. To that end, we used Zinpyr1, a Zn fluorophore that is able to bind to Zn found in complex with an AA (Fig. S1b). To investigate Zn uptake from ZnAA in vitro, and to characterize the uptake pathways, we used the human intestinal cell line Caco-2 since the intestine is the first tissue confronted with large quantities of Zn. Caco-2 cells were incubated for 30 min with ZnCl_2_ solution (50 μM) or ZnAAs delivering an equivalent of 50 μM Zn. The mean intracellular Zn concentration per cell was determined by Zinpyr-1 fluorescence intensity. Zinpyr-1 is a membrane-permeant fluorescent sensor for Zn with a high specificity and affinity (Kd = 0.7 ± 0.1 nM) (Burdette et al. 2001). However, Zinpyr-1 does not only detected “free” but also weakly protein bound Zn and the pool of Zn detectable by Zinpyr-1 is measured. Further, the content of Zn in ZnAA preparations was determined by AAS (Fig. S1c). As ZnAA solutions were prepared according to the MW of ZnAAs from powder that may contain traces of moisture and bisulfate acting as Zn donor in the production process, final concentrations measured for ZnAAs were slightly lower as calculated and the concentration of ZnAA used in the experiments adjusted to deliver an equivalent of Zn compared to Zn-delivery by ZnCl_2_ solution (50 μM).

As expected, treatment of cells with ZnCl_2_ solution led to a significant increase in Zn concentrations (ANOVA on ranks, H = 94.125, *p* < 0.001, Dunn’s multiple comparison test: Control vs. ZnCl_2_ *p* < 0.0001). Similarly, ZnGlu, ZnLys/Glu and ZnLys/Glu/Met significantly increased intracellular Zn (Fig. 1b) (Control vs. ZnGlu *p* = 0.0028, Control vs. ZnLys *p* = 0.0741; Control vs. ZnLys/Glu *p* < 0.0001; Control vs. ZnLys/Glu/Met *p* = 0.0144). ZnCl_2_ solution showed stronger effects compared to ZnMet and ZnLys (ZnCl_2_ vs. ZnLys *p* = 0.0138; ZnCl_2_ vs. ZnMet *p* = 0.0004). 120 min after application, ZnCl_2_ solution and all ZnAAs increased intracellular Zn levels (one-way ANOVA, F_6.69_ = 21.619, *p* < 0.0001, Bonferroni post hoc analysis: Control vs. ZnCl_2_, ZnGlu, ZnLys, ZnMet, ZnLys/Glu, ZnLys/Glu/Met *p* < 0.0001), although ZnMet increased intracellular Zn levels the least (Fig. 1c).

While application of Glu and Lys alone did not alter intracellular Zn levels compared to untreated controls, the application of Met alone, in turn, resulted in a significant increase in intracellular Zn (Fig. 1d), possibly due to secondary effects (one-way ANOVA, F = 165.893, *p* < 0.001, Bonferroni post hoc analysis: Control vs. ZnCl_2_, Met *p* < 0.0001; ZnCl_2_ vs. Met *p* < 0.0001). However, the uptake of free Zn provided via ZnCl_2_ solution was not affected by the presence of Glu, Lys or Met (Fig. 1e) (one-way ANOVA, F = 53.22, *p* < 0.0001, Bonferroni post hoc analysis: Control vs. ZnCl_2_, ZnCl_2_ + Glu, ZnCl_2_ + Lys, ZnCl_2_ + Met, *p* < 0.0001).

Next, we investigated whether the uptake of Zn provided by ZnAAs is inhibited by co-application of the corresponding AA. In contrast to the application of ZnCl_2_, a significant inhibitory effect on the increase in intracellular Zn levels after application of ZnGlu, ZnLys, and ZnMet was observed in presence of Glu, Lys and Met, respectively (Fig. 1f–h) (Fig. 1f: one-way ANOVA, F = 157, *p* < 0.0001, Bonferroni post hoc analysis: Control vs. ZnGlu *p* < 0.0001; Control vs. ZnGlu + Glu *p* = 0.027; Control vs. ZnLys *p* < 0.0001; Control vs. ZnMet *p* < 0.0001; Fig. 1g Lys: one-way ANOVA, F = 7.841, *p* < 0.0001, Bonferroni post hoc analysis: Control vs. ZnLys *p* = 0.007, ZnLys vs. ZnLys + Lys (150) *p* = 0.003, ZnLys vs. ZnLys + Lys (200) *p* < 0.0001, ZnLys vs. ZnLys + Lys (200) *p* < 0.0001; Fig. 1g Glu: one-way ANOVA, F = 91.44, *p* < 0.0001, Bonferroni post hoc analysis: Control vs. ZnGlu *p* < 0.0001, Control vs. ZnGlu + Glu (10) *p* < 0.0001, Control vs. ZnGlu + Glu (50) *p* < 0.0001, Control vs. ZnGlu + Glu (150) *p* < 0.0001, ZnGlu vs. ZnGlu + Glu (100) *p* < 0.0001, ZnGlu vs. ZnGlu + Glu (150) *p* < 0.0001, ZnGlu vs. ZnGlu + Glu (200) *p* < 0.0001). The inhibition of Zn uptake after application of ZnAAs via surplus of the corresponding AA follows a competitive manner as the increase in intracellular Zn is reduced in a concentration dependent manner by co-application of the AA (Fig. 1f). Thus, it is possible that Zn uptake, depending on the source (ZnCl_2_ or ZnAAs) is mediated differently in Caco-2 cells. Even 120 min after application of ZnCl_2_ solution and ZnAAs, similar mechanisms were detected (Fig. S2a, b) hinting towards stability of ZnAA complexes.

### Uptake of Zn from ZnAAs is only partially affected by uptake antagonists

**Fig. 2:**
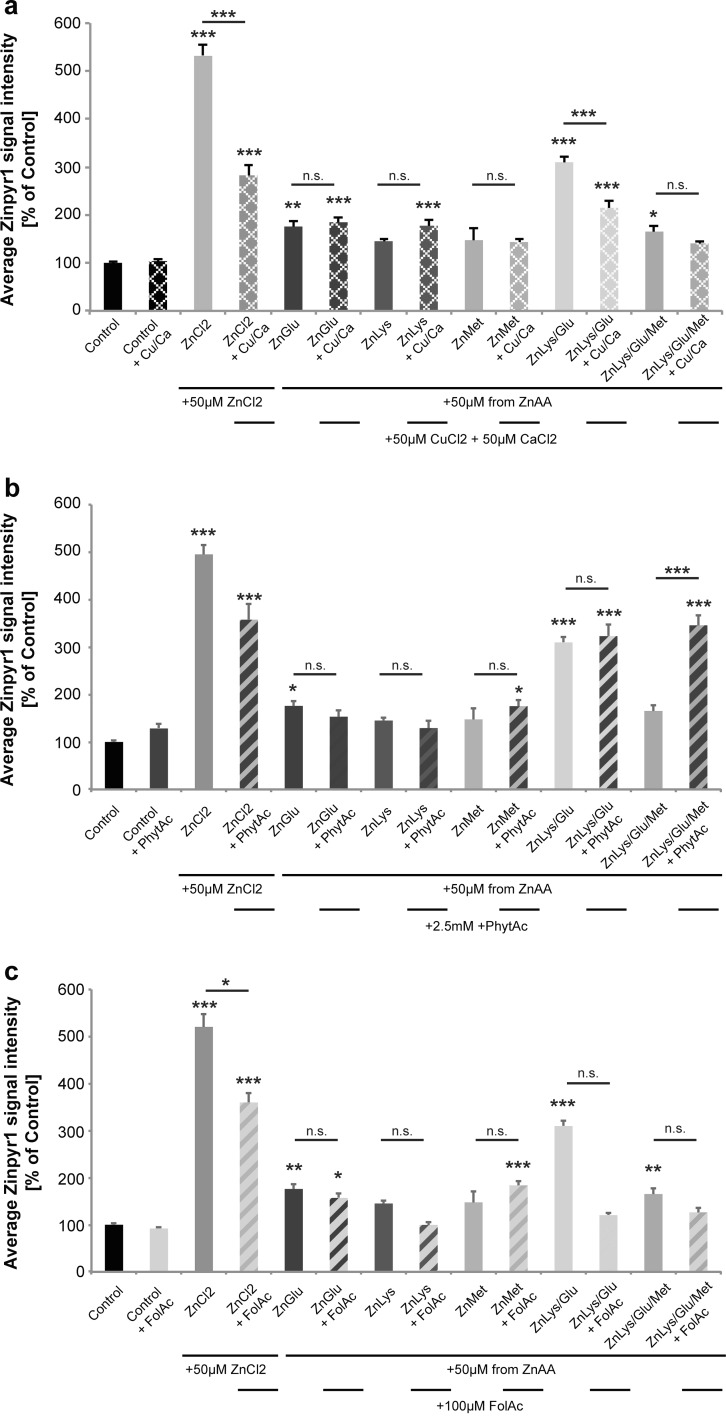
Effect of antagonistic factors on uptake of Zn from ZnAAs in Caco-2 cells. Zinpyr-1 fluorescence intensity of Caco-2 cells. **a** Application of Zn uptake antagonists calcium (provided as CaCl_2_) and copper (provided as CuCl_2_) together with ZnCl_2_ solution and ZnAAs. A significant inhibitory effect on Zn uptake from ZnCl_2_ solution is seen. No significant inhibition of the uptake of Zn delivered by ZnGlu, ZnLys, ZnMet, and ZnLys/Glu/Met was observed. The uptake of ZnLys/Glu was significantly inhibited. **b** Co-application of Zn uptake antagonist phytic acid (PhytAc). Co-application of ZnCl_2_ and phytic acid leads to significantly less increase in intracellular Zn levels compared to cell treated only with ZnCl_2_ solution. No significant antagonistic effect was observed upon co-application of phytic acid and ZnGlu, ZnLys, ZnMet, ZnLys/Glu, and ZnLys/Glu/Met. Uptake of ZnLys/Glu/Met was higher in presence of phytic acid. Phytic acid was used in a molar ratio Zn:PhytAc = 1:50 (*n* = 10). **c** Co-application of Folic acid (FolAc). Significantly less Zn uptake via ZnCl_2_ solution (FolAc was used with 100 μM concentration) is observed. A significant inhibitory effect on the uptake of ZnLys, ZnLys/Glu, and ZnLys/Glu/Met was observed. Only ZnGlu and ZnMet are unaffected, although ZnMet did not significantly increase intracellular Zn levels within 30 min (*n.s.* not significant)

The presence of antagonists of Zn uptake such as increased amounts of Ca and Cu (Fig. 2a), phytic acid (PhytAc) (Fig. 2b), and folic acid (FolAc) (Fig. 2c) did not alter the significant increase in intracellular Zn levels 30 min after application of ZnAAs in most cases, but Ca and Cu, as well as phytic acid and folic acid affected Zn uptake from ZnCl_2_. The presence of 50 μM CuCl_2_ and 50 μM CaCl_2_ significantly reduced Zn uptake from the ZnCl_2_ solution (Fig. 2a; ANOVA on ranks, H = 142.288, *p* < 0.0001; Dunn’s multiple comparisons test: Ctrl vs. ZnCl_2_, *p* < 0.0001; ZnCl_2_ vs. ZnCl_2_ + Ca/Cu, *p* < 0.0001). The presence of Ca and Cu in contrast did not affect the uptake of Zn from ZnGlu, ZnLys, ZnMet, and ZnLys/Glu/Met (ZnGlu vs. ZnGlu + Ca/Cu, *p* > 0.999; ZnLys vs. ZnLys + Ca/Cu, *p* > 0.9999; ZnMet vs. ZnMet + Ca/Cu, *p* > 0.999; ZnLys/Glu/Met vs. ZnLys/Glu/Met + Ca/Cu, *p* > 0.9999). Although, as seen previously, 30 min after treatment with ZnLys and ZnMet, no significant increase in intracellular Zn levels was observed, the presence of Ca and Cu did not affect intracellular Zn concentrations.

The application of 2.5 mM phytic acid similarly reduced the significant increase in intracellular Zn when Zn was added in the form of ZnCl_2_ (Fig. 2b; ANOVA on ranks, H = 139.436, *p* < 0.001; Dunn’s multiple comparisons test: Ctrl vs. ZnCl_2_, *p* < 0.0001). In contrast, phytic acid did not significantly impair the increase caused by ZnGlu and ZnLys/Glu, and uptake of ZnLys/Glu/Met (ZnGlu vs. ZnGlu + PhytAc, *p* > 0.9999; ZnLys/Glu vs. ZnLys/Glu + PhytAc, *p* > 0.9999; ZnLys/Glu/Met vs. ZnLys/Glu/Met + PhytAc, *p* > 0.9999). Again, ZnLys and ZnMet did not increase intracellular Zn levels significantly within 30 min of application, but phytic acid also did not affect Zn levels in these conditions (ZnLys vs. ZnLys + PhytAc, *p* > 0.9999; ZnMet vs. ZnMet + PhytAc, *p* > 0.9999).

The presence of folic acid (Fig. 2c) also affected the significant increase in intracellular Zn delivered by ZnCl_2_ solution (Fig. 2c; ANOVA on ranks, H = 134.578, *p* < 0.0001; Dunn’s multiple comparisons test: Ctrl vs. ZnCl_2_, *p* < 0.0001; ZnCl_2_ vs. ZnCl_2_ + FolAc, *p* = 0.0482). Again, no significant effect of FolAc on the increase in intracellular Zn levels caused by application of ZnGlu was seen (ZnGlu vs. ZnGlu + FolAc, *p* = 0.0537). Folic acid had no inhibitory effect on Zn in the form of ZnLys, ZnLys/Glu, and ZnLys/Glu/Met (ZnLys vs. ZnLys + FolAc, *p* > 0.9999; ZnLys/Glu vs. ZnLys/Glu + FolAc, *p* = 0.0541; ZnLys/Glu/Met vs. ZnLys/Glu/Met + FolAc, *p* > 0.9999). ZnLys and ZnMet did not increase intracellular Zn levels significantly within 30 min of application, but folic acid also did not affect Zn levels significantly after application of ZnMet and even lead to a slight increase.

Thus, the significant intracellular increase in Zn levels after application of ZnCl_2_ solution was impaired by co-application of all antagonists tested. In contrast, the significant increase after application of ZnGlu was unaffected by any of the antagonists. All ZnAAs seem to be protected from inhibitory effects caused by phytic acid and folic acid.

### Zn from ZnAAs is taken up by AA transporters in human enterocytes

**Fig. 3:**
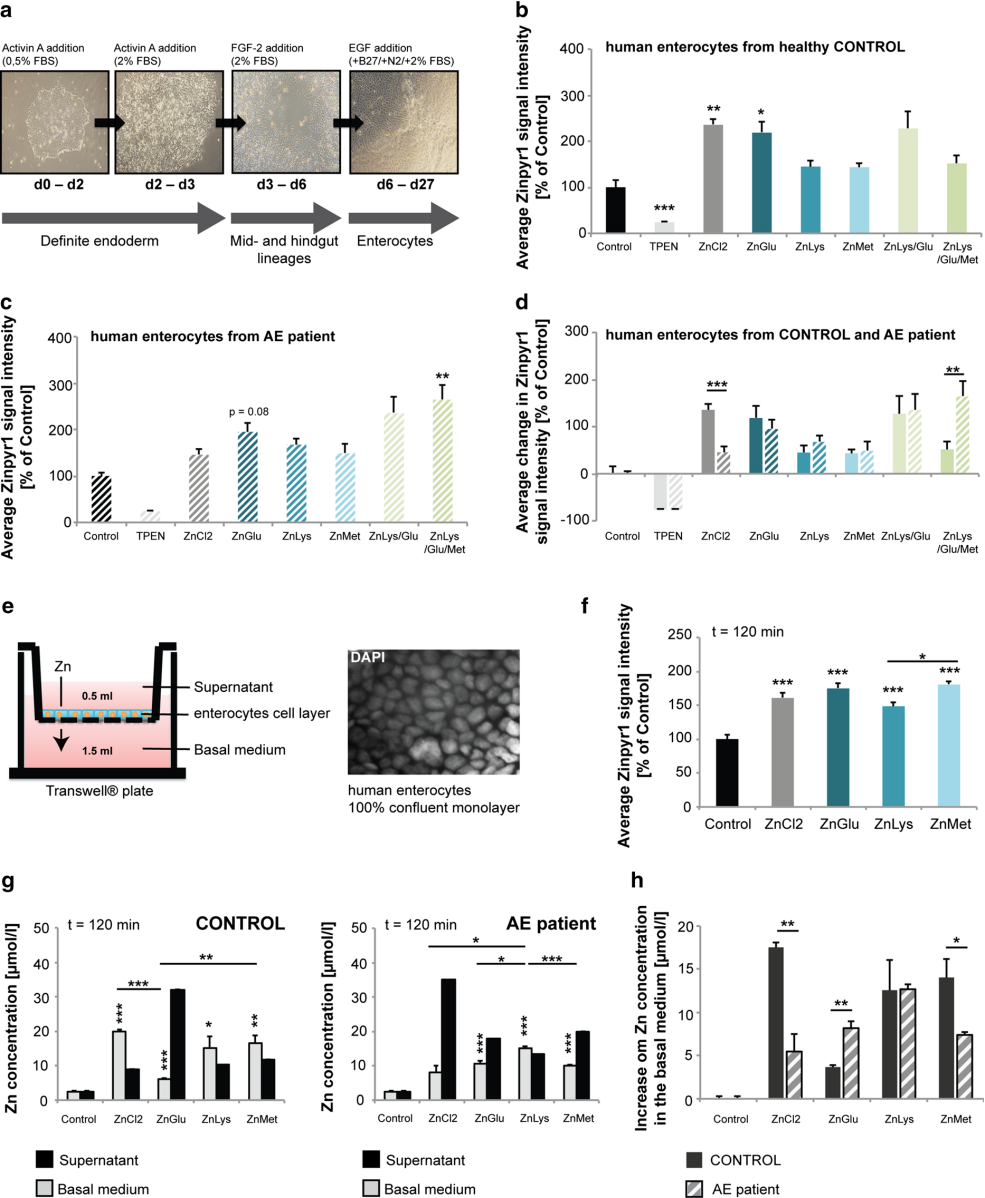
Zn uptake in human enterocytes differentiated from healthy controls and patients with *Acrodermatitis enteropathica*. **a** Overview of the protocol for generation of enterocytes from hIPSC of a healthy control and AE patient. **b**, **c** Enterocytes were exposed to ZnCl_2_ solution (50 μM) or ZnAAs delivering an equivalent of 50 μM Zn for 30 min. Treatment with the Zn chelator TPEN confirmed the specificity of the Zinpyr-1 signal. **b** Intracellular Zn levels in enterocytes of the healthy control are significantly increased after treatment with ZnCl_2_ solution and ZnGlu. ZnLys, ZnMet, ZnLys/Glu and ZnLys/Glu/Met did not significantly increase intracellular Zn within 30 min after application. **c** Intracellular Zn levels in enterocytes of the *AE* patient did not show a significant increase after treatment with ZnCl_2_. Application of ZnLys/Glu/Met (significant) and ZnGlu (as trend) leads to an increase in intracellular Zn levels. **d** A significant difference in uptake of Zn from ZnCl_2_ solution between enterocytes from Control and the AE patient was detected. Uptake of ZnAAs is not significantly lower in enterocytes from the *AE* patient compared to the Control. Uptake from ZnLys/Glu/Met was significantly higher in cells from the AE patient. **e** Enterocytes differentiated from hIPSC were plated on the filter of a transwell^®^ system. ZnCl_2_ solution or ZnAAs were applied to the supernatant. After 120 min, the supernatant and the basal medium were removed. Cells on the filter were grown until they were 100% confluent and are visualized by DAPI staining of cell nuclei. **f** After incubation with ZnCl_2_ or ZnAAs, a significant increase in Zinpyr-1 staining in cells (*n* = 10) was detected. **g** Left: Using cells from the Control, all treatments resulted in a significant increase of Zn concentration in the basal medium (measured by AAS). The absorption of ZnGlu was significantly less compared to ZnCl_2_ solution and ZnMet. Right: Using cells from the *AE* patient, incubation with ZnCl_2_ solution did not result in a significant increase of Zn concentration in the basal medium compared to untreated control cells, while application of all ZnAAs significantly increase the Zn concentration in the basal medium. The absorption of ZnLys was significantly higher compared to ZnCl_2_ solution, ZnGlu and ZnMet. **h** Comparing the healthy control and the AE patent, significant differences were detected in the absorption of Zn from ZnCl_2_ solution and after application of ZnGlu and ZnMet

To further investigate whether ZnAAs are taken up by AA transporters before dissociation of Zn from the AA, we performed uptake experiments using enterocytes differentiated from human induced pluripotent stem cells (hiPSC) from a healthy control and a patient with *AE* (Fig. 3a–d). The underlying cause of *AE* in this patient was identified by genome sequencing and the results revealed compound heterozygous mutations 192 + 19G > A and 599C > T (AA sequence Pro200Leu) in the hZIP4 gene. Thus, in this case, Zn uptake is inhibited by a genetic mutation in the major Zn importer. Differentiated cells from control and patient were identified as enterocytes by the expression of the marker proteins Sucrase-Isomaltase (SI) and Peptidase 1 (SLC15A1), and CDX2 and Villin (Fig. S3a, b). No difference in differentiation efficacy was found between control and patient.

After differentiating hiPSC to enterocytes (Fig. 3a), we again exposed the cells from a healthy control and *AE* patient to ZnCl_2_ solution (50 μM) or ZnAAs delivering an equivalent of 50 μM Zn. Treatment with the Zn chelators TPEN confirmed the specificity of the Zinpyr-1 signal. 30 min after treatment, we measured the intracellular Zn levels by Zinpyr-1 staining (Fig. 3b–d). The results show that intracellular Zn levels in enterocytes of the healthy control are significantly increased after treatment with ZnCl_2_ solution as observed before in Caco-2 cells (Fig. 3b, ANOVA on ranks, H = 52.424, *p* < 0.0001; Dunn’s multiple comparisons test: Ctrl vs. ZnCl_2_, *p* = 0.0015). A significant increase in intracellular Zn was also observed upon treatment with ZnGlu (Ctrl vs. ZnGlu, *p* < 0.0001), while ZnLys, ZnMet and ZnLys/Glu/Met did not significantly increase intracellular Zn within 30 min after application and the increase of ZnLys/Glu was statistically visible only as trend. All measured Zinpyr-1 fluorescence intensities were significantly different from the TPEN control.

In contrast, enterocytes differentiated from iPSCs from the *AE* patient did not show a significant increase in intracellular Zn after treatment with ZnCl_2_ solution (Fig. 3c, d) (Fig. 3c: ANOVA on ranks, H = 45.97, *p* < 0.0001; Dunn’s multiple comparisons test: Ctrl vs. ZnCl_2_, *p* > 0.9999). Compared to enterocytes differentiated from iPSCs from the Control, the uptake of Zn from ZnCl_2_ solution was significantly less (Fig. 3d: ZnCl_2__Control_ vs. ZnCl_2 AE_, *p* < 0.0001). This is in line with the expectation that Zn delivered by ZnCl_2_ solution is mainly taken up by Zip4, which is severely functionally impaired in the *AE* patient. In contrast, ZnAAs are taken up by enterocytes differentiated from the *AE* patient and significantly increased intracellular Zn levels in case of ZnLys/Glu/Met (Fig. 3c: Ctrl vs. ZnLys/Glu/Met, *p* = 0.0071), and ZnGlu tended to increase intracellular Zn levels (Fig. 3c: Ctrl vs. ZnGlu, *p* = 0.0787). All measured Zinpyr-1 fluorescence intensities were significantly different from the TPEN control. In contrast to uptake by ZnCl_2_ solution, uptake of ZnAAs is not significantly different between enterocytes from Control and the *AE* patient (Fig. 3d: ZnGlu_Control_ vs. ZnGlu_AE_, *p* = 0.4665; ZnLys_Control_ vs. ZnLys_AE_, *p* = 0.2688; ZnMet_Control_ vs. ZnMet_AE_, *p* = 0.8083; ZnLys/Glu_Control_ vs. ZnLys/Glu_AE_, *p* = 0.8772) with exception of ZnLys/Glu/Met which significantly increased intracellular Zn level in cells from the AE patient 30 min after application (Fig. 3d: ZnLys/Glu/Met_Control_ vs. ZnLys/Glu/Met_AE_, *p* = 0.0055).

Enterocytes differentiated from control iPSC also display similar uptake of compounds and ZnCl_2_ solution compared to Caco-2 cells in presence of uptake antagonists (Fig. S3c). 30 min after treatment with ZnCl_2_ solution or ZnGlu, a significant increase in intracellular Zinpyr-1 fluorescence was detected (ANOVA on ranks, H = 36.342, *p* < 0.0001; Dunn’s multiple comparisons test: Ctrl vs. ZnCl_2_, *p* < 0.0001; Ctrl vs. ZnGlu, *p* = 0.0103). While uptake of Zn from ZnCl_2_ solution was significantly impaired by Ca, Cu, and phytic acid and tended to be impaired by folic acid (ANOVA on ranks, H = 36.8, *p* < 0.0001; Dunn’s multiple comparisons test: ZnCl_2_ vs. ZnCl_2_ + Ca/Cu, *p* < 0.0001; ZnCl_2_ vs. ZnCl_2_ + PhytAc, *p* < 0.0001; ZnCl_2_ vs. ZnCl_2_ + FolAc, *p* = 0.0804), uptake of ZnGlu was not significantly affected by presence of antagonists (ANOVA on ranks, H = 1.515, *p* = 0.6787). Although application of ZnLys and ZnMet did not result in significant increases in intracellular Zinpyr-1 fluorescence within 30 min, a significant alteration of Zn levels was only observed for ZnLys in presence of FolAc (ANOVA on ranks, H = 28.51, *p* < 0.0001; Dunn’s multiple comparisons test: ZnLys vs. ZnLys + FolAc, *p* = 0.0003) and for ZnMet in presence of Ca/Cu (ANOVA on ranks, H = 14.68, *p* < 0.0001; Dunn’s multiple comparisons test: ZnLys vs. ZnLys + FolAc, *p* < 0.0001).

### Zn from ZnAA is absorbed in similar quantity compared to free Zn

Therefore, next, to study not only uptake but actual absorption, we used a transwell^®^ system (Fig. 3e). Enterocytes differentiated from human iPS cells were plated on the filter of a transwell insert and grown until they were 100% confluent (Fig. 3e). To control for gaps, FITC Dextran was applied to some wells and the increase in fluorescence in the basal medium controlled (Fig. S4). ZnCl_2_ solution or ZnAAs were applied to the supernatant. After 120 min, the supernatant and the basal medium (Fig. 3e) were removed and the transport of Zn through the layer of cells was determined by measuring the Zn concentration of supernatant and basal medium (Fig. 3g, h). The results show that 120 min after treatment, the intracellular Zn concentration was significantly elevated after incubation with ZnCl_2_ solution or ZnAAs (Fig. 3f) (one-way ANOVA, F_4.49_ = 19.316, *p* < 0.0001, Bonferroni post hoc analysis: Control vs. ZnCl_2_, ZnGlu, ZnLys, ZnMet *p* < 0.0001). In enterocytes differentiated from iPSC from the Control, incubation with ZnCl_2_ solution (50 μM Zn) or ZnAAs (50 μM Zn) resulted in a significant increase of Zn concentration in the basal medium compared to untreated control cells (one-way ANOVA, F = 53.716, *p* < 0.0001, Bonferroni post hoc analysis: Control vs. ZnCl_2_, *p* < 0.0001, Control vs. ZnGlu, *p* = 0.0006; Control vs. ZnLys, *p* = 0.0238; Control vs. ZnMet *p* = 0.0029). However, the absorption of ZnGlu was significantly less compared to ZnCl_2_ solution and ZnMet (ZnCl_2_ vs. ZnGlu, *p* < 0.0001; ZnGlu vs. ZnMet *p* = 0.0085). In enterocytes differentiated from iPSC from the *AE* patient, incubation with ZnCl_2_ solution (50μM Zn) did not result in a significant increase of Zn concentration in the basal medium compared to untreated control cells (one-way ANOVA, F = 30.691, *p* < 0.0001, Bonferroni post hoc analysis: Control vs. ZnCl_2_, *p* = 0.0544). In contrast, application of ZnAAs (50 μM Zn) resulted in a significant increase of Zn concentration in the basal medium (Control vs. ZnGlu, *p* = 0.0008; Control vs. ZnLys, *p* < 0.0001; Control vs. ZnMet *p* < 0.0001). The absorption of ZnLys was significantly higher compared to ZnCl_2_ solution, ZnGlu and ZnMet (ZnCl_2_ vs. ZnLys, *p* = 0.0259; ZnGlu vs. ZnLys *p* = 0.0103; ZnMet vs. ZnLys *p* = 0.0008) (Fig. 3g).

Thus, while uptake of Zn into cells is faster by ZnCl_2_ compared to most ZnAAs after 30 min, uptake is similar after 120 min, which is reflected in similar levels of absorption after 120 min with slight advantage for ZnLys. Comparing enterocytes from the healthy control and the AE patent (Fig. 3h), significant differences can be detected (one-way ANOVA, F = 42.013, *p* < 0.0001). Post hoc analyses reveal a difference in absorption of Zn from ZnCl_2_ solution (ZnCl_2 Control_ vs. ZnCl_2 AE_, *p* = 0.0047) that was significantly less in cells from the AE patient. Further differences were detected after application of ZnGlu, which was significantly better absorbed in cell from the AE patient (ZnGlu_Control_ vs. ZnGlu_AE_, *p* = 0.0073), and ZnMet (ZnMet_Control_ vs. ZnMet_AE_, *p* = 0.0373) that showed less absorption in cell from the AE patient compared to cells from the healthy control.

### Uptake of Zn by ZnAAs does not affect Zn and AA importer expression or localization

Homeostasis of intracellular Zn concentrations, under physiological conditions, is maintained in cells by specific regulatory pathways. An increase in intracellular Zn, for example, leads to Zn binding to the metal transcription factor-1 (MTF1) that translocates into the nucleus in a Zn-bound state. There, MTF-1 binds to specific sequences, so called metal response elements (MREs) in the promoter region of target genes, among them ZnT1. This increases the mRNA expression levels of ZnT1 and subsequently protein numbers. Given that ZnT1 is a Zn exporter, this increase leads to a reduction of intracellular Zn levels maintaining initial Zn homeostasis. We therefore quantified the expression levels and subcellular localization of selected candidate genes such as ZIP2, ZIP4 and ZnT1 on mRNA and protein level. Both, ZIP2 and ZIP4 have been shown to regulate Zn uptake into enterocytes. The level of mRNA abundance and subcellular localization of ZIP4 were reported to change in response to Zn levels (Dufner-Beattie et al. 2003).

**Fig. 4:**
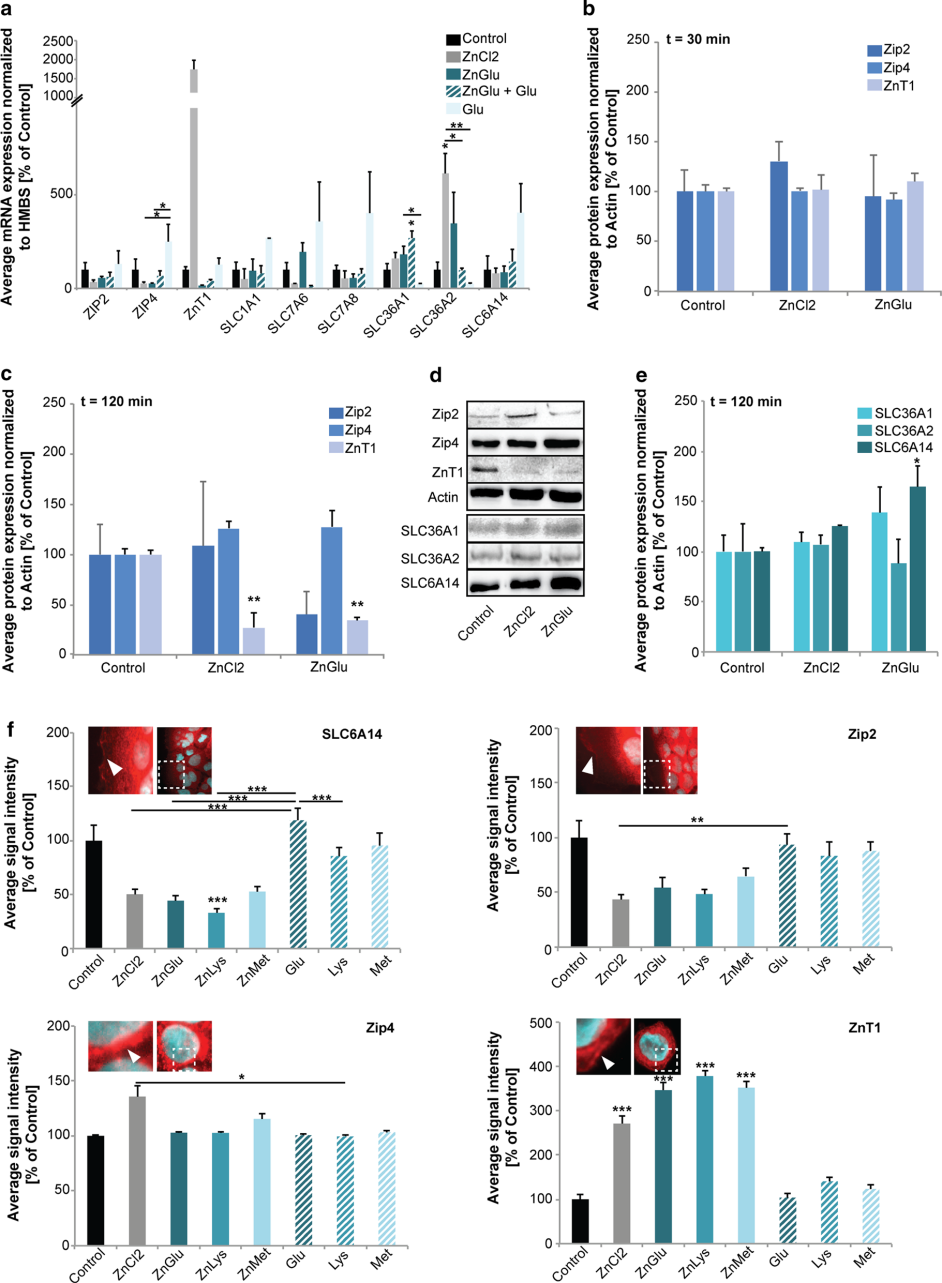
Physiological responses to Zn uptake in Caco-2 cells. **a** Quantification of gene expression levels using qRT-PCR. The average mRNA expression (Δct value) normalized to HMBS is shown compared to untreated (control) cells. Cells were treated with ZnCl_2_ (50 μM), ZnGlu (delivering an equivalent of 50 μM), ZnGlu and Glu (200 μM), or Glu (200 μM) alone. No significant changes were detected for the Zn transporters ZIP2 and ZnT1. Expression of ZnT1 shows high dynamics and a clear trend towards an up-regulation. ZIP4 levels are significantly different between cells treated with Glu compared to ZnCl_2_ and ZnGlu treatment. Amino acid transporters (SLC1A1, SLC7A6, SLC7A8 and SLC6A14) are unaffected by the treatments. SLC36A1 is significantly up-regulated after treatment with ZnGlu + Glu and shows significantly different expression in response to ZnGlu vs. Glu alone. SLC36A2 shows significantly increased expression after treatment with ZnCl_2_ and ZnGlu (as a trend) (*n* = 3 per condition). **b**–**d** Expression of Zip2, Zip4 and ZnT1 on protein level using Western Blot. The integrated density of 3 immunoreactive bands per condition was measured. **b** 30 min after application of ZnCl_2_ and ZnGlu, no significant regulation can be detected on protein level. **c** After 120 min, no alterations are detected regarding total Zip2 and Zip4 levels. A significant decrease in total cell ZnT1 concentration is observed after treatment with ZnCl_2_ or ZnGlu. **d** Exemplary western blot bands for the evaluation of Zip2, Zip4, ZnT1 and SLC36A1, SLC36A2, and SLC6A14 protein levels after 120 min. **e** The AA transporter SLC6A14 shows an up-regulation on total protein level after 120 min only after treatment with ZnGlu. No significant difference is detected for SLC36A1 and SLC36A2. **f** Immunocytochemistry was performed on Caco-2 cells (*n* = 10 per condition) and the fluorescence intensity of SLC6A14, Zip2, Zip4 and ZnT1 signals measured 120 min after treatment. A significant decrease of cell surface SLC6A14 signals after treatment with ZnLys but not the other ZnAAs is detected. Application of Lys alone does not elicit these alterations. Glu treatment slightly enhances SLC6A14 surface localization. For Zip2, no significant differences are detected in cell surface location, although a trend towards a reduction after ZnCl_2_ treatment and treatment with ZnAAs is seen (significant for ZnCl_2_ vs. Glu). No significant differences are detected in cell surface location of Zip4. The levels of ZnT1 at the cell surface were significantly increased after treatment with both ZnCl_2_ solution and ZnAAs, despite the decrease in total protein levels (**c**)

Alternatively, Zn complexes with AAs might bypass this system and might be taken up as well as exported, in part, via AA transporters. Thus, we additionally analyzed selected candidate genes such as AA transporters SLC1A1, SLC7A6, SLC7A8, SLC6A14, SLC36A1 and SLC36A2 on mRNA and protein level (Fig. 4). SLC7A6, SLC7A8, SLC36A1 and SLC3A2 are sodium-independent AA transporters expressed in enterocytes and Caco-2 cells, mediating the uptake of a wide range of AAs including Met, Lys and Glu. SLC1A1 and SLC6A14 are sodium-dependent AA transporters.

Our results reveal no significant changes of Zn transporters compared to controls in response to ZnCl_2_ solution or ZnAAs, although expression levels of ZnT1 are highly increased after administration of ZnCl_2_ (Fig. 4a). The AA transporters (SLC1A1, SLC7A6, SLC7A8 and SLC6A14) are unaffected by any of the treatments (Fig. 4a: one-way ANOVA, *p* > 0.05). However, SLC36A2 is significantly up-regulated on mRNA level after treatment with ZnCl_2_ solution (one-way ANOVA, F_4.14_ = 7.608, *p* = 0.004, Bonferroni post hoc analysis: Control vs. ZnCl_2_ *p* = 0.021).

To test whether differences might occur on protein level rather than in gene expression, we quantified the expression of Zip2, Zip4 and ZnT1 using Western Blot analysis (Fig. 4b–d). 30 min after application of ZnCl_2_ and ZnGlu, no significant regulation can be detected on protein level (Fig. 4b, one-way ANOVA, ZIP2: F_2.8_ = 0.2954 *p* = 0.7545; ZIP4: F_2.8_ = 0.4382 *p* = 0.6643; ZnT1: F_2.8_ = 0.1858 *p* = 0.8351). After 120 min, no changes were detected in Zip2 (one-way ANOVA, F_2.8_ = 0.754 *p* = 0.51) and Zip4 (F_2.8_ = 2.201 *p* = 0.192), and a down-regulation of total cellular ZnT1 protein was found for both treatment groups (one-way ANOVA, F_2.8_ = 19.088 *p* = 0.003; Bonferroni post hoc analysis: Control vs. ZnCl_2_ *p* = 0.004; Control vs. ZnGlu *p* = 0.007) (Fig. 4c, d). The AA transporter SLC6A14 showed a significant increase only in response to ZnGlu and not to ZnCl_2_ (Fig. 4d, e) (one-way ANOVA, F_2.8_ = 7.102 *p* = 0.026; Bonferroni post hoc analysis: Control vs. ZnCl_2_ *p* = 0.584; Control vs. ZnGlu *p* = 0.029).

In addition to alterations in expression levels, the localization of transporters at the membrane might change. Thus, we additionally performed immunocytochemistry on treated Caco-2 cells and evaluated immuno-reactive signals at the cell surface of Zip2, Zip4, ZnT1 and SLC6A14 (Fig. 4f). The results show that a significant change occurs in SLC6A14 signals (one-way ANOVA, F_7.79_ = 46.604 *p* < 0.0001). Bonferroni post hoc analysis reveals that the SLC6A14 fluorescence intensity at the membrane after treatment with ZnLys significantly decreases (Control vs. ZnLys *p* < 0.0001). Interestingly, treatment with Lys alone, but also treatment with ZnCl_2_ solution did not induce this change (Control vs. Glu *p* = 0.055; Control vs. Lys *p* = 0. 204; Control vs. ZnCl_2_ *p* = 0. 077). Zip2 membrane expression was not altered between the treatment groups and control, but a significant difference was found between treatment with ZnCl_2_ solution and Glu (one-way ANOVA, F_7.79_ = 20.720 *p* = 0.003; Bonferroni post hoc analysis: ZnCl_2_ vs. Glu *p* = 0.002). Membrane Zip4 levels were significantly altered (ANOVA on ranks, H = 21.52 *p* = 0.0031; Dunn’s post hoc test: ZnCl_2_ vs. Lys *p* = 0.034). However, no significant alterations after application of ZnCl_2_ or ZnAAs compared to Control were observed. ZnT1 levels at the plasma membrane were significantly increased after both treatment with ZnCl_2_ solution and ZnGlu, ZnLys and ZnMet (one-way ANOVA, F_7.79_ = 62.766 *p* < 0.0001; Bonferroni post hoc analysis: Control vs. ZnCl_2_ *p* < 0.0001; Control vs. ZnGlu, ZnMet and ZnLys *p* < 0.0001).

Thus, taken together, application of ZnAAs does not elicit a homeostatic down-regulation of Zn importers on mRNA or protein level. However, treatment of Caco-2 cells with ZnAAs leads to an up-regulation of cell surface ZnT1 leading to Zn export. In the gastrointestinal tract, this export would be directed to the blood circulation. In addition, application of ZnAAs also does not lead to major compensatory regulations of AA transporters. Thus, Zn uptake from ZnAAs over time is not reduced, possibly bypassing homeostatic mechanisms that diminish Zn uptake upon chronic exposure to high Zn levels.

## Discussion

Zn is absorbed from our dietary sources and/or supplements in the proximal small intestine, either the distal duodenum or proximal jejunum (Krebs et al. 1998; Lee et al. 1989) and released in the blood stream via transporters. However, various agents present in the diet can decrease Zn absorption (Cousins 2010). To prove this in an in vivo situation, we used mice and fed them a special diet containing either adequate Zn concentrations, inadequate Zn concentrations, or adequate Zn concentrations in presence of increased levels of Zn uptake antagonists such as phytic acid, Ca/Fe, and folic acid. Control animals displayed normal Zn levels in whole-blood in a magnitude as reported before (Grabrucker et al. 2016). As expected mice on a Zn deficient diet showed a significant reduction of Zn levels in the blood after 9 weeks. As whole-blood Zn was investigated, this reduction does not only reflect decreased Zn levels of a fast exchanging pool of plasma Zn, but indicated a systemic Zn deficiency affecting intracellular Zn levels in several tissues. An important finding is that a similar Zn deficiency is produced in animals fed the control diet with adequate Zn supply but with increased levels of Zn uptake antagonists. In addition, genetic mutations in Zn transporters such as Zip4 in *AE*, may significantly lower Zn uptake.

Today, suboptimal Zn intake that might also lead to secondary nutritional problems is present not only in many developing countries but also in industrial nations (Lönnerdal 2000). Supplementation with Zn has, among others, effects on linear growth (Brown et al. 1998), the immune system (Shankar and Prasad 1998), and pregnancy outcome (Goldenberg et al. 1995). Intriguingly, among individuals with Autism Spectrum Disorders (ASD), the incidence rate of Zn deficiency has been reported to be significantly higher compared to age matched healthy control subjects (Pfaender and Grabrucker 2014). In the light of these findings, maintaining adequate Zn status during pregnancy and early development might be a promising approach to prevent cognitive and neurobehavioral deficits later in life. However, in most cases and especially in industrialized nations, the cause of suboptimal Zn absorption is not an inadequate dietary intake of Zn, but the presence of antagonists in the diet. Therefore, it is necessary to investigate the efficacy of different Zn compounds in their ability to deliver Zn despite the presence of inhibitory factors or abnormalities in Zn transporters.

Here, we investigated the route of absorption of ZnAA compounds. Our results show that Zn delivered by ZnAAs is taken up into cells in a similar magnitude as Zn delivered as free Zn (by Zn salts) after two hours, although uptake is slower. Similar results were reported before using CuAAs, where the uptake of CuAAs was lower compared to inorganic Cu solution, but the amino acid complex forms facilitated Cu absorption in Caco-2 cells. The enhanced absorption of CuAA appeared to be mediated by amino acid transporters (Gao et al. 2014). Bath application of 50 μM Zn led to an approx. 3 fold increase in intracellular Zn levels after two hours. In this study, changes in Zn levels induced by ZnCl_2_ or ZnAA supplementation are shown relative to controls. Measuring absolute Zn concentrations using Zinpyr-1 fluorescence signal intensities is difficult to perform, as several components, such as buffering proteins in the medium of cells that contains fetal bovine serum (FBS), introduce variables that may lead to a non-linear realtionship of Zn and fluorescence intensity. Further, quantitative measurements of Zn concentrations using fluorophores such as Zinpyr-1 are difficult, as not only free but also protein bound Zn is detected to some extent, and comparisons of relative values between ZnCl_2_ and ZnAAs may be more meaningful.

It was shown that with increasing amounts of Zn in a diet, the amount of Zn absorbed decreases (Lönnerdal 2000; Sandström and Cederblad 1980). This reduction in fractional absorption of zinc at higher doses most likely is due to the saturation of Zn transporters. For example, in humans after intestinal perfusion with solutions with increasing concentration of Zn, first a linear increase in Zn absorption was observed. However, at higher concentrations, the rates leveled out (Lee et al. 1989).

Intriguingly, in cells from an *AE* patient, no impairment in the absorption of ZnAAs was seen in contrast to ZnCl_2_. We could show that, at least in part, ZnAA are taken up via AA transporters. Thus, uptake of Zn from ZnAAs is not impaired in cells from an *AE* patient with mutated ZIP4 gene, but inhibited by increased levels of AA competing with ZnAAs for AA transporters. Although uptake may also be mediated by other transporters such as DMT-1, it is unlikely that DMT-1 transports ZnAAs and is inhibited by AAs. Further, the structure of the ZnAAs makes it unlikely that they function as ionophores. AAs generally show poor membrane permeability (Chakrabarti 1994) and ZnAAs are structurally not drastically different from the corresponding AAs.

While we could not detect major changes in mRNA and total protein levels of zinc importers Zip2 and Zip4 after treatment with ZnAAs, similar to exposure to ZnCl_2_ solution, levels of membrane bound ZnT1 exporters were significantly increased as homeostatic control mechanism of cells to reduce increased intracellular Zn levels (Cousins 2010). However, we could not detect a reduction of AA transporters in response to ZnAA. Given that Zn importer and AA importer levels are unaffected by exposure to increased levels of ZnAA, it is likely that the uptake of Zn by ZnAA is a process that will not be compensated by homeostatic regulation of AA transports and given the abundance of AA transporters will not be easily saturated. It might even be possible to increase Zn levels beyond physiological concentrations using ZnAAs.

Zn is likely to predominantly be transported as “free” Zn via a saturable, specific transport mechanism such as Zn importers from the ZIP family (Sandström 1992). This, however, makes the “free” Zn susceptible for competition with other metals and dietary ligands that may considerably lower the concentration of “free” Zn. Zn delivery by ZnAA, in particular ZnGlu, which is among the most stable ZnAAs tested, is less affected by uptake inhibitors compared to Zn delivery by “free” Zn from ZnCl_2_ solution. Especially high levels of Ca and Cu were not able to inhibit uptake of Zn via ZnGlu and ZnLys/Glu/Met in vitro.

Although it is unlikely that the level of Cu in the diet or in a supplement would reach the levels used in this study in vivo, an interaction of Ca and Cu has been described in experimental animals, albeit at very high ratios. Most likely, Cu and Zn compete on cellular level for binding partners necessary for absorption such as metallothioneins or metal transporters. However, the Cu/Zn interaction is at present poorly understood. The data obtained through our studies argues for a possible competition already at the level of cellular uptake as the increase of intracellular Zn by application of “free” Zn was significantly decreased in presence of high Ca and Cu concentrations, but uptake of ZnGlu and other ZnAAs seemed unaffected.

Delivery of Zn by ZnGlu, ZnLys/Glu and ZnLys/Glu/Met was also not affected by the presence of high levels of phytic acid. Animal experiments have shown that the absorption of Zn is inversely correlated to the concentration of phytic acid in the diet (Lönnerdal et al. 1988). The phosphate groups in phytic acid form insoluble complexes with Zn, and phytate-bound Zn will be excreted in the feces, thereby causing an inhibitory effect on Zn absorption (O’Dell 1969; Vohra and Kratzer 1964). Phytate is present in staple foods like cereals, corn and rice, and the removal or reduction of phytate by methods such as phytase treatment or fermentation markedly improves Zn absorption. In case of ZnAA, it is possible that Zn will not be as accessible as “free” Zn and thus less complex formation with phytic acid will occur.

Folic acid has been reported to increase fecal Zn excretion, which might indicate an inhibitory effect on Zn absorption (Ghishan et al. 1986; Simmer et al. 1987; Krebs 2000). Intriguingly, pregnant women are encouraged to supplement folic acid in their diet, as a lack of folic acid has been associated with birth defects such as *spina bifida*. However, recent studies suggest that excessive amounts of folate might increase the risk for the occurrence of ASD (Raghavan et al. 2016). One might speculate that the underlying patho-mechanism could be a prenatal Zn deficiency that has been shown to induce autism-like behavior in mice (Grabrucker et al. 2014, 2016; Grabrucker 2014).

Uptake of most ZnAAs tested was affected by the presence of folic acid, which might argue for a possible indirect interaction of folic acid with processes such as Zn homeostasis. However, we observed no significant influence of increased levels of folic acid on Zn delivery by ZnGlu. ZnGlu therefore might be an interesting compound if Zn should be supplemented together with folic acid.

Our result supports the need for a careful assessment of bioavailability of Zn in the human diet, as dietary habits may change and required daily dosages might need to be adjusted to prevent Zn deficiencies. Especially during pregnancy, consumption of supplements containing high folic acid and Ca and Fe concentrations may create the need for higher Zn intake to ensure maintenance of physiological Zn levels and possibly reduce the risk for neurodevelopmental disorders in the offspring.

Taken together, we conclude that Zn can be enriched in cell systems and animals using ZnAAs. The uptake of ZnAAs might be slower, however, uptake and absorption reaches a similar quantity compared to “free” Zn over time. Most importantly, the advantage of ZnAAs lies in their ability to utilize less saturable pathways for uptake and in the feature that ZnAA compounds are protected from antagonistic factors in the diet to some extent. Therefore, they might be a superior supplement in cases of genetic defects in pathways responsible for uptake of “free” Zn, such as mutations in Zn transporters as seen in *AE*, or in case of diets with high concentrations of uptake antagonists and in combination with other supplements delivering Cu or folic acid for example.

## Materials and methods

### Materials, chemicals and reagents

Zinpyr-1 was purchased from Sigma Aldrich. Primary antibodies were purchased from Synaptic Systems (ZnT1), Abcam (Zip2 (1:1000), Zip4 (1:125), SLC6A14), Acris (Zip4 (1:200)), Thermo Fisher (SLC36A1 (1:500)), Invitrogen (SLC36A2 (1:500)), and Sigma Aldrich (β-Actin, (1:1000)). Secondary HRP conjugated antibodies were purchased from Dako (anti-rabbit 1:1000) and Dianova (anti-mouse 1:10,000). DMEM, DMEM/F12 GlutaMAX, RPMI advanced, Non-essential amino acids (NEAA), B27, N2, FBS and Antibiotic–Antimycotic were acquired from Gibco. Unless otherwise indicated, all other chemicals were of analytical grade and obtained from Sigma-Aldrich.

*ZnAAs*—Zinc amino acid complexes (ZnAAs) (Fig. S1a) were obtained from Zinpro Corporation (Eden Prairie, MN USA). ZnGlu, ZnMet, ZnLys and, to utilize as many amino acid transporters as possible (cationic, anionic and hydrophobic transporters), combinations were used. We chose lysine since it has an active transporter. It also has a stability constant greater than that of methionine in metal complexes (Furia 1975). The complex with Zn is between the carboxyl group and the alpha nitrogen. The side group may increase the stability but is not needed for binding. ZnAA structures with the complex between the acid and amine groups forming a 5 membered ring. The complexes are known to survive the stomach acid and get absorbed in animals based on the results of several studies where animals were supplemented with ZnAA or ZnAA in addition to inorganic Zn and significant differences were detected compared to supplementation with Zn salt (Nayeri et al. 2014). However, the stability of of ZnAAs in low stomach pH has not yet been directly determined.

ZnAA compounds were obtained as powder and solutions were freshly prepared for each experiment. The calculated MW for a ZnAA was taken into account to prepare the solutions.

### Caco-2 colorectal adenocarcinoma cell line

Caco-2 cells (American Type Culture Collection (ATCC^®^ HTB-37™)) were used between passage 20 and 43. The cells were maintained in 175-cm^2^ flasks in Dulbecco’s minimal essential medium (DMEM) supplemented with 10% fetal bovine serum, 1% non-essential amino acids (NEAA), 1% l-Glutamine, and 1% Antibiotic–Antimycotic 100× (Invitrogen). Cells were grown to 100% confluence on a 24 well plate or 10 cm petri dish. Treatment was performed on DIV 10, as Caco-2 cells differentiate spontaneously in culture to mature intestinal enterocytes within 21d, and we detected expression of Zn and AA transporters and enterocyte like morphology after 10 d in culture with no change until DIV 21, except for a decrease of ZIP4 in Caco-2 cells older than 10 DIV (Fig. S5a–c).

### Animals

Female C57BL/6JRj mice were purchased from Janvier labs. Mice (8 weeks of age) were habituated for two weeks (receiving the same diet). Subsequently, mice received a special laboratory diet (ssniff GmbH, Germany; Supplementary data 1) with access to food and water ad libitum for 9 weeks. Animals on Zn deficient diet received distilled water; all other groups had access to tap water. Three animals per group were analyzed. All animal experiments were performed in compliance with the guidelines for the welfare of experimental animals issued by the Federal Government of Germany and by the local ethics committee (Ulm University) ID Number: 1257.

### Generation of iPSC from a patient with Acrodermatitis enteropathica

After informed consent, keratinocytes were obtained from plucked human hair and plated on matrigel diluted 1:10 in EpiLife (Gibco). Plated hair was cultivated in MEF conditioned medium supplemented with 10 ng/ml FGF2 (Cell Guidance Systems), 20 ng/ml Ascorbic Acid (Sigma-Aldrich) and 10 µM Rho-associated kinase (ROCK) inhibitor (Ascent Scientific) and medium changed to Epilife supplemented with 10 µM ROCK inhibitor after keratinocyte outgrowth. IPSC lines were reprogrammed from human hair keratinocytes via lentiviral transduction as previously described with a multicistronic vector published by Warlich et al. (2011) (Linta et al. 2012). iPSC were cultured in mTeSR1 medium (Stemcell Technologies) on matrigel (BD Biosciences) coated 6-well plates at 37 °C, 5% CO_2_ and 5% O_2_.

### Stem cell-differentiation into enterocyte-like cells

The differentiation was performed using a protocol by Iwao et al. (2014). Human induced pluripotent stem cell lines between passages 15 and 20 were grown to 70% confluence in mTeSR1 stem cell medium. Differentiation to definite endoderm was initiated by changing the medium to RPMI advanced supplemented with 2 mM GlutaMAX, 0.5% FBS, 100 ng/ml Activin A (Cell Guidance Systems) and 1% Antibiotic–Antimycotic. After 48 h, cells were grown in RPMI advanced supplemented with 2 mM GlutaMAX, 2% FBS, 100 ng/ml Activin A and 1% Antibiotic–Antimycotic for 24 h. The medium was changed to DMEM/F12 GlutaMAX containing 2% FBS, 250 ng/ml FGF-2 (Cell Guidance Systems) and 1% Antibiotic–Antimycotic for 96 h. Subsequently cells were treated with 10 μM ROCK Inhibitor (Ascent Scientific) for 1 h, detached with TrypLE and plated on Matrigel coated 12-well plates. The cells were cultured in DMEM/F12 + GlutaMAX, 2% FBS, 1% NEAA, 2% B27, 1% N2, 20 ng/ml EGF (Invitrogen), and 1% Antibiotic–Antimycotic. B27 and N2 were purchased from GIBCO. Medium was changed every 3 days. The cells were used after 3 weeks in culture (d21). Cells were grown to 100% confluence on a 24 well plate or a 12 well Corning^®^ Costar^®^ Transwell^®^ plate with 3 μm pore Polyester (PET) membrane insert and also used at d21. Confluency and leakage through gaps in the cell layer we controlled by microscopy and application of FITC Dextran, respectively. FITC Dextran fluorescence was determined using a fluorimeter (Jenway 62 series) and UV table.

### Immunocytochemistry

After fixation in 4% Paraformaldehyde/4% Sucrose at room temperature for 15 min, the cells were washed with PBS and permeabilized with 0.2% Triton X-100 in PBS for 5 min twice. Subsequently, blocking solution (BS; 10% FBS in PBS) was applied at room temperature for 1 h. Cells were incubated with the primary antibodies at 4 °C over night. After washing 3× with PBS, the cells were incubated with Alexa Fluor conjugated secondary antibody diluted 1:1000 in BS at room temperature for 1 h. After washing 2× with PBS and 1× with sterile Millipore water, cells were mounted with ProLong^®^ Gold antifade reagent with DAPI. Fluorescence images were obtained an upright Axioscope microscope equipped with a Zeiss CCD camera (16 bits; 1280 × 1024 ppi) using Axiovision software (Zeiss) and analyses of integrated densities was performed with ImageJ 1.50 g.

### Measurement of trace metal concentrations

For fluorescent Zn-staining of cultured cells, growth medium was discarded and the cells were washed with PBS. Coverslips were incubated with a solution of 50 μM Zinpyr-1 (Burdette et al. 2001) in PBS for 1 h at RT. The mean intracellular Zn concentration per cell was determined by Zinpyr-1 fluorescence intensity. Images were obtained using an Axioscope microscope and evaluated with ImageJ 1.50 g. The Zn-concentration of solutions and blood was measured by flame atomic absorption spectrometry (AAS) at the Department of Clinical Chemistry (ZE klinische Chemie) of the University Hospital Ulm using a PinAAcle 900T from Perkin Elmer and in the workgroup of Prof. Rink, Institute of Immunology, University Hospital Aachen using a Perkin Elmer AAnalyst 800 instrument. All reagents for atomic absorption were of appropriate quality for trace element analysis (TraceSelect, Fluka). Samples were diluted in ultrapure water containing 0.2% (v/v) HNO_3_ and quantified with a standard curve between 0 and 1 mg l^−1^ zinc in 0.2% (v/v) HNO_3_, measuring the absorption at 213.9 nm (slit 0.7 nm). A reference samples spiked with defined concentrations of Zn were routinely analyzed to ensure reliable quantification. Whole blood from mice was collected in Lithium-Heparin Microvettes (Sarstedt) and stored at −20 °C until further analysis.

### Cell lysis and RNA preparation

Cells were harvested and resuspended in 600 µl RIPA buffer, and kept for 30 min on ice. Lysate was cleared from debris by centrifugation (14,000 rpm, 15 min) and stored at −80 °C with Protease Inhibitor. Total RNA was isolated from cell-pellets resuspended in RLT buffer with the RNeasy Mini Kit according to the manufacturer’s protocol. All of the optional purification steps were performed and RNA eluted with sterile RNAse free water.

### qRT-PCR

First strand synthesis and quantitative real-time-PCR amplification were performed in a one-step, single-tube format using the QuantiFast™ SYBR Green RT-PCR kit from Qiagen according to the manufacturer’s protocol in a total volume of 20 µl and the Qiagen QuantiTect Primer Assays: Hs_CDX2_1_SG (QT00037807), Hs_VIL1_1_SG (QT00095837), Hs_SLC39A4_va.1_SG (QT01022637), Hs_HMBS_1_SG (QT00014462), Hs_SLC39A2_1_SG (QT00213388), Hs_SLC30A1_1_SG (QT00096320), Hs_SLC1A1_1_SG (QT00065303), Hs_SLC7A6_1_SG (QT00052248), Hs_SLC7A8_1_SG (QT00006384), Hs_SLC36A1_1_SG (QT00029281), Hs_SLC36A2_1_SG (QT00070875), Hs_SLC6A14_1_SG (QT00087542). The qRT-PCR was set up with the following program (Reverse Transcription, 50 °C, 10′, 1 cycle; Denaturation, 95 °C, 5′, 1 cycle; [Denaturation, 95 °C, 10′′, combined annealing/extension, 60 °C, 30′′], 40 cycles; Melting curve analysis, 60–95 °C, 90′′ (first step, elevation of 1 °C/step), 5′′ (following steps); Cooling, 4 °C). Thermal cycling and fluorescent detection were performed using the Rotor-Gene Q real-time PCR machine (model 2-Plex HRM) (Qiagen). The SYBR Green I reporter dye signal was measured. Resulting data were analyzed using the HMBS gene as an internal standard to normalize transcript levels. Cycle threshold (ct) values were calculated by the Rotor-Gene Q Software (version 2.0.2). All quantitative real-time PCR reactions were run in technical triplicates for each of the 3 patient and 2 control cell lines and mean ct-values for each reaction were taken into account for calculations.

### Protein biochemistry

To obtain homogenate from cultures, cells were lysed and homogenized in lysis buffer (150 mM NaCl, 1% Triton X-100, 50 mM Tris–HCl, pH 8.0) containing protease inhibitor (Roche). Cell debris and nuclei were removed by centrifugation at 3200 rpm for 10 min. Protein concentration was determined by Bradford protein assay. Proteins were separated by SDS-PAGE and blotted onto nitrocellulose membranes (GE Healthcare). Immunoreactivity was visualized using HRP-conjugated secondary antibodies and the SuperSignal detection system (Pierce).

### Statistic

Statistical analysis was performed with GraphPad Prism 7. Data are shown as mean ± SEM. For comparisons, analysis of variance (ANOVA) was performed followed by post hoc tests for within group comparisons (Dunn’s test, or Bonferroni test). For comparisons of two independent groups, student’s *t*-tests was used. Statistically significant differences are indicated in the figures by *p ≤ 0.05, **p ≤ 0.01 and ***p ≤ 0.001.

*qRT PCR quantification*—Relative quantification is based on internal reference genes to determine virtual mRNA levels of target genes. Cycle threshold (ct) values were calculated by the Rotor-Gene Q Software (version 2.0.2). Ct values were transformed into virtual mRNA levels according to the formula: virtual mRNA level = 10 * ((ct_(target)_ − ct_(standart)_)/slope of standard curve).

*Western blot quantification*—Evaluation of bands from Western blots (WBs) was performed using ImageJ. Three independent experiments were performed and blots imaged using a MicroChemi Imaging System from Biostep. The individual bands were selected and the integrated density was measured. All WB bands were normalized to β-Actin and the ratios averaged.

*Immunocytochemistry*—For cell culture experiments, 10 cells of each condition were imaged. The signal intensity of immuno-reactive signals was quantified using ImageJ software.

## Electronic supplementary material

Below is the link to the electronic supplementary material.

Supplementary material 1 (PDF 61 kb)

Supplementary material 2 (TIFF 1074 kb)

Supplementary material 3 (TIFF 1643 kb)

Supplementary material 4 (TIFF 9336 kb)

Supplementary material 5 (TIFF 3518 kb)

Supplementary material 6 (TIFF 3212 kb)
