# Supplementary material for: Characterization of zinc amino acid complexes for zinc delivery in vitro using Caco-2 cells and enterocytes from hiPSC
Source: Biometals. 2017 Jul 17;30(5):643–61. doi: 10.1007/s10534-017-0033-y (PMC5646115; doi:10.1007/s10534-017-0033-y)
Supplement: Supplementary file 1 — Supplementary material 1 (PDF 61 kb) [file 10534_2017_33_MOESM1_ESM.pdf]

Ingredient		Diet 1	Diet 2	Diet 3
		Control	zinc deficient	zinc inhibitor
Crude protein	%	19.3	19.3	19.3
Crude fat	%	5.2	5.2	5.2
Crude fibre	%	5.4	5.4	5.4
Crude ash	%	6.5	6.5	7.5
Sugar	%	2.5	2.5	4.5
Dextrin	%	5.6	5.6	1.3
NfE	%	53.5	53.5	51.2
Lysine	%	1.23	1.23	1.23
Methionine	%	0.76	0.76	0.76
Met & Cys	%	1.01	1.01	1.01
Threonine	%	0.81	0.81	0.81
Tryptophan	%	0.25	0.25	0.25
ME, physiol. Fuel value	MJ/kg	14.2	14.2	13.8
ME - pig	MJ/kg	14	14	13.6
**Calcium**	%	0.72	0.72	**1.13**
Phosphorus	%	0.55	0.55	0.69
**Phytate**	%	4.5	4.5	**9.5**
Sodium	%	0.32	0.32	0.32
**Magnesium**	%	0.16	0.16	0.16
Potassium	%	0.6	0.6	0.6
**Iron**	mg/kg	113	113	**503**
Manganese	mg/kg	40	40	40
**Zinc**	mg/kg	41	**19**	41
Copper	mg/kg	13	13	13
Selenium	mg/kg	0.24	0.24	0.24
Iodine	mg/kg	1.54	1.54	1.54
Vitamin A	IU/kg	15000	15000	15000
Vitamin D3	IU/kg	1500	1500	1500
Vitamin E	mg/kg	54	54	54
Vitamin K	mg/kg	8	8	8
Vitamin B1	mg/kg	18	18	18
Vitamin B2	mg/kg	17	17	17
Vitamin B6	mg/kg	18	18	18
Vitamin B12	ug/kg	100	100	100
Niacin	mg/kg	78	78	78
Panthothenic acid	mg/kg	59	59	59
**Folic acid**	mg/kg	0.7	0.7	**1.9**
Biotin	ug/kg	680	680	680
Choline	mg/kg	1350	1350	1350
