# Supplementary figures and images for: Characterization of zinc amino acid complexes for zinc delivery in vitro using Caco-2 cells and enterocytes from hiPSC
Source: Biometals. 2017 Jul 17;30(5):643–61. doi: 10.1007/s10534-017-0033-y (PMC5646115; doi:10.1007/s10534-017-0033-y)

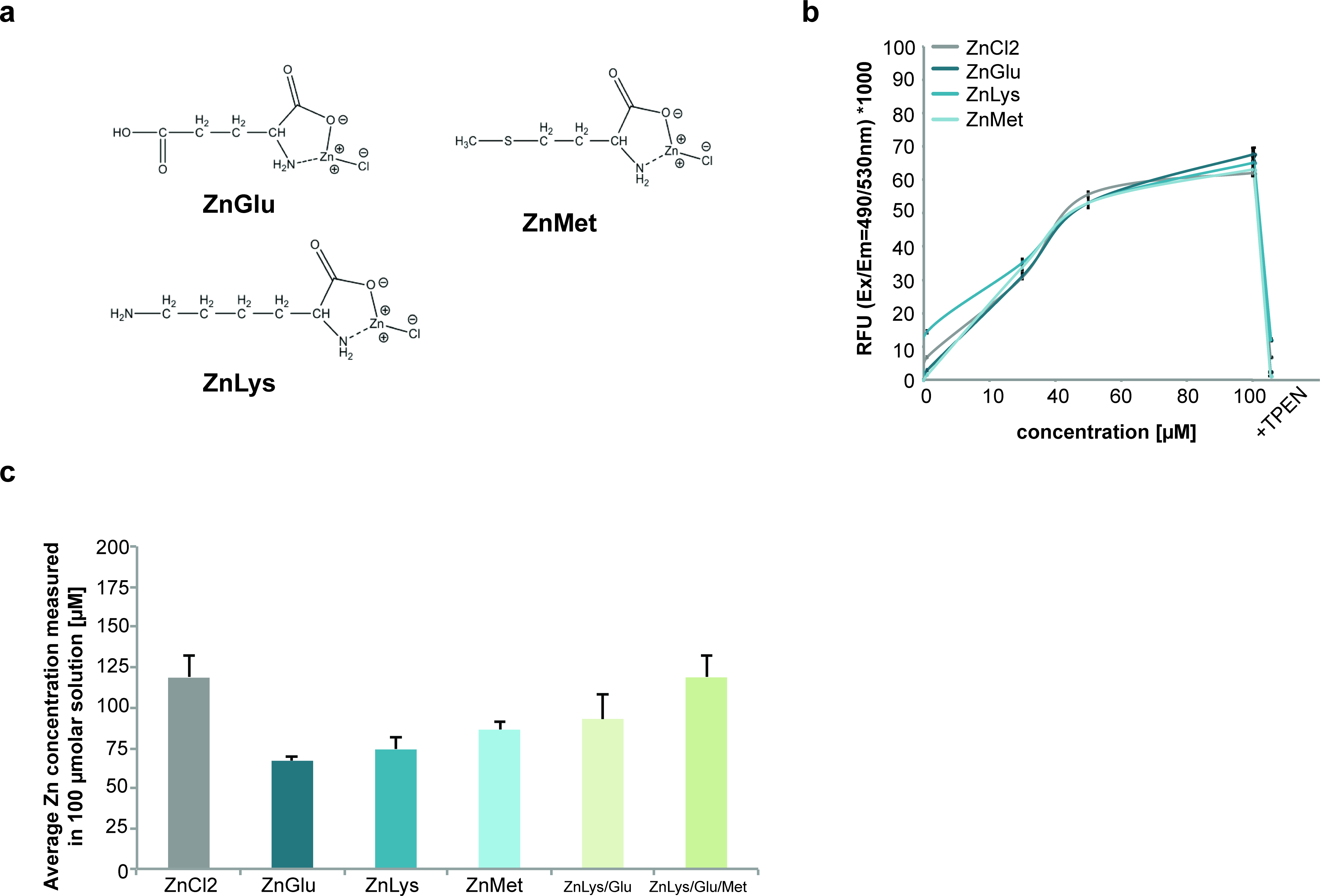

Supplement: Supplementary file 2 — Supplementary material 2 (TIFF 1074 kb) [file 10534_2017_33_MOESM2_ESM.tif]

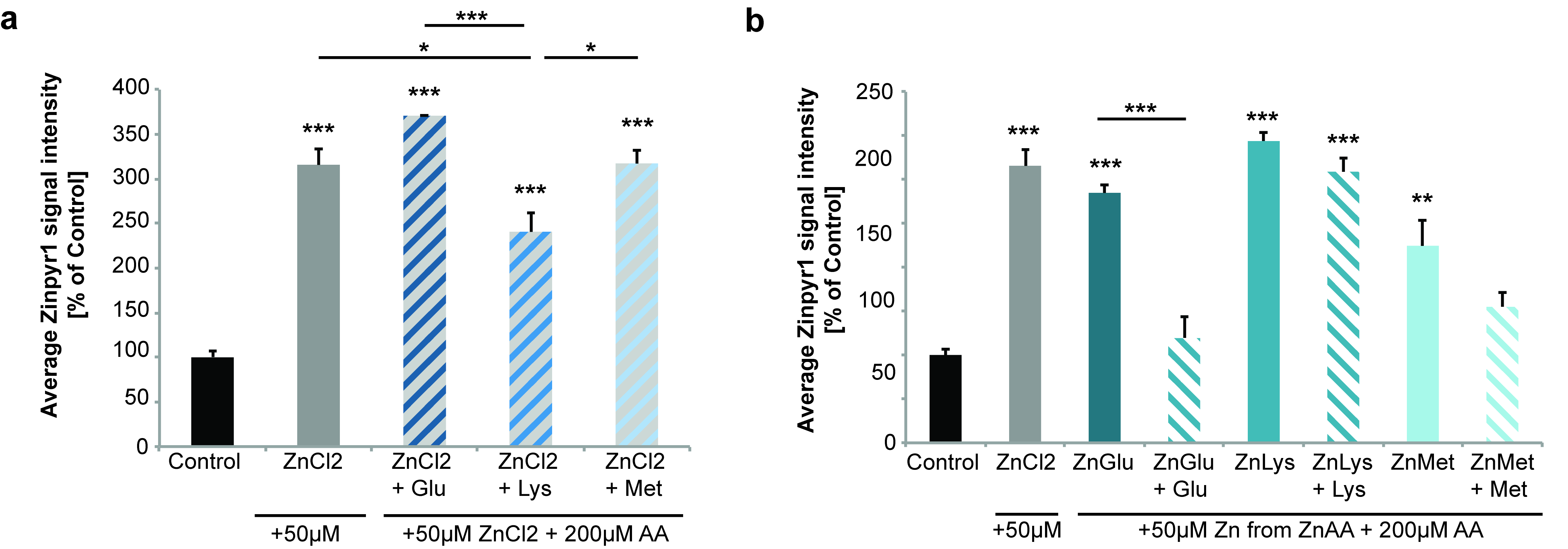

Supplement: Supplementary file 3 — Supplementary material 3 (TIFF 1643 kb) [file 10534_2017_33_MOESM3_ESM.tif]

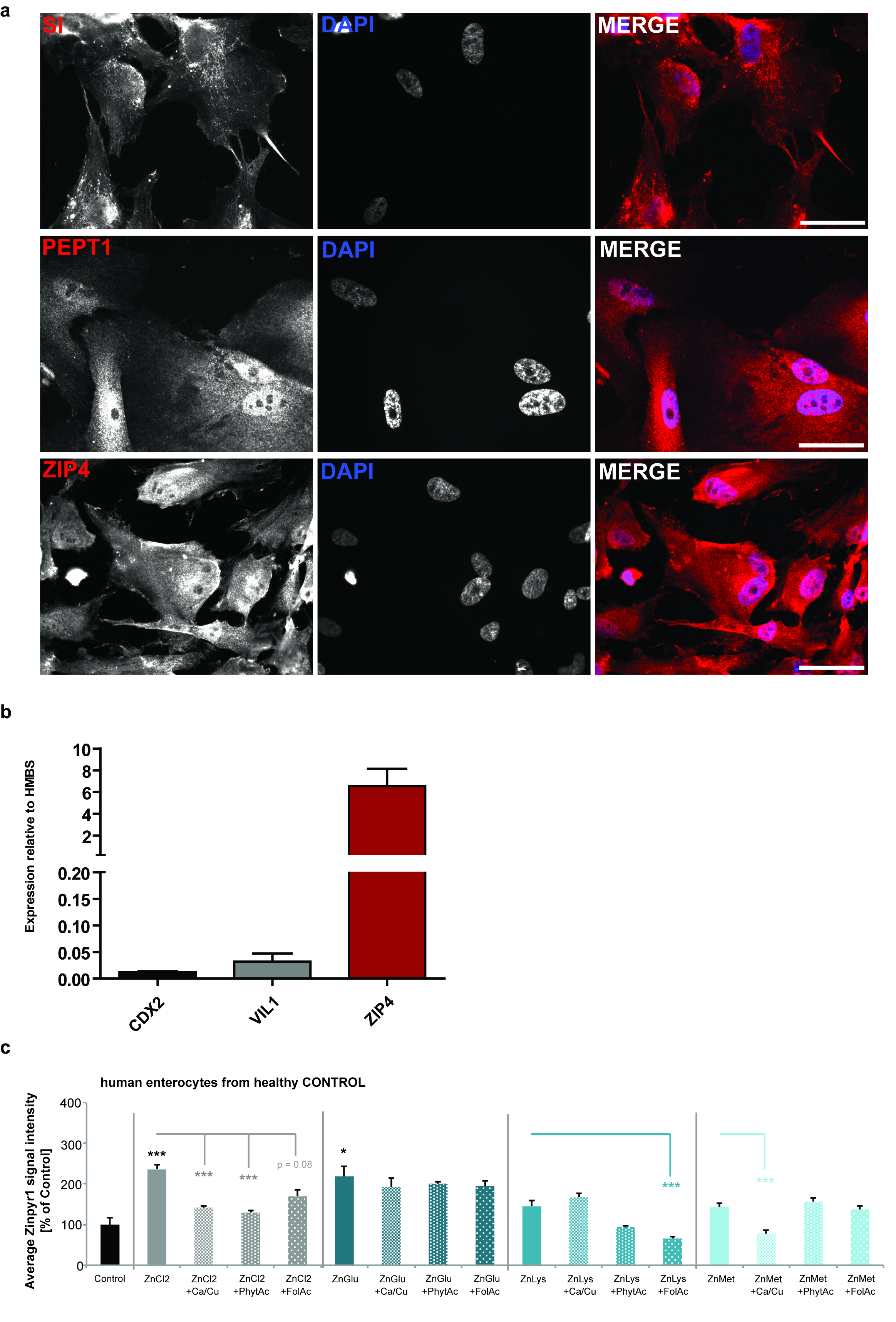

Supplement: Supplementary file 4 — Supplementary material 4 (TIFF 9336 kb) [file 10534_2017_33_MOESM4_ESM.tif]

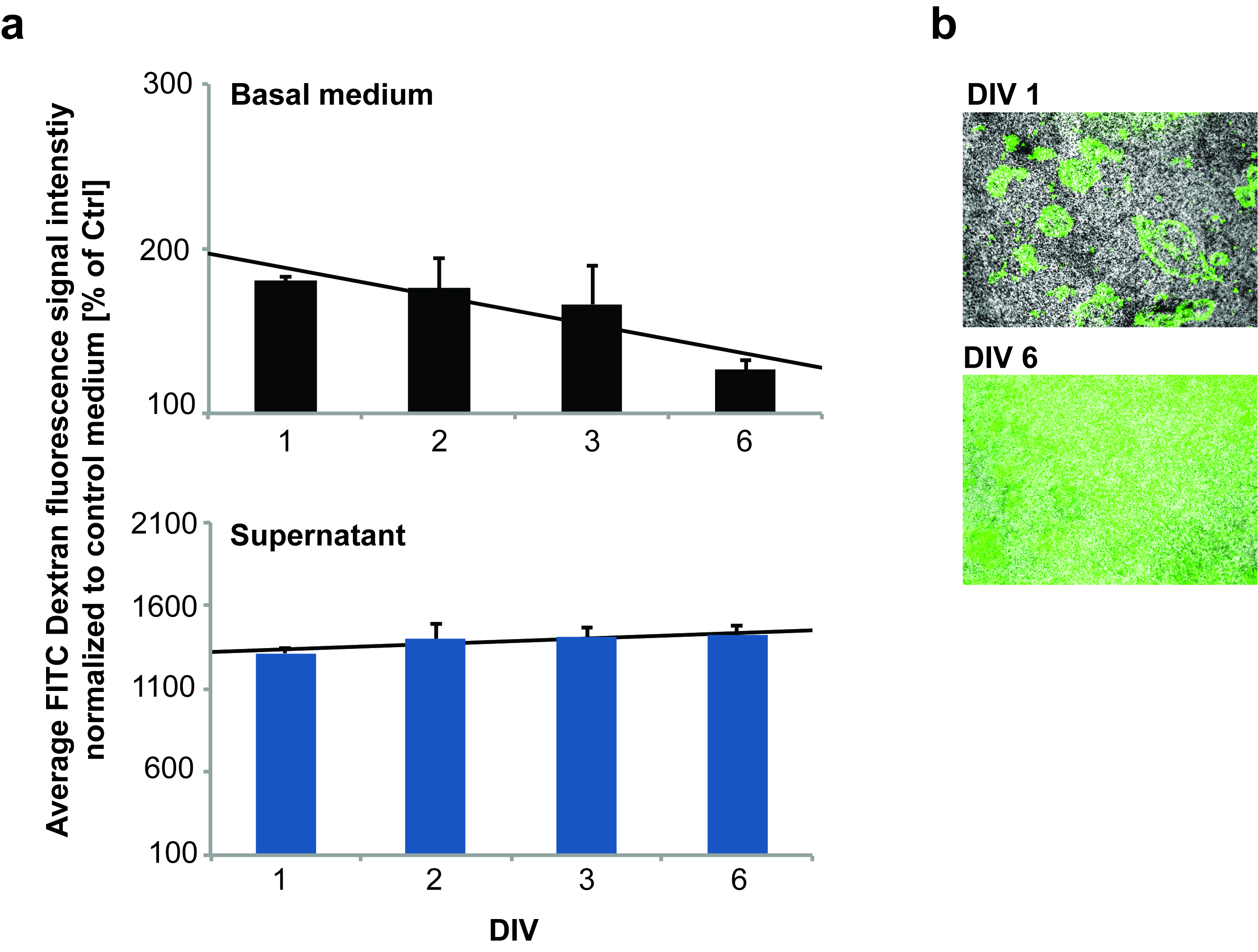

Supplement: Supplementary file 5 — Supplementary material 5 (TIFF 3518 kb) [file 10534_2017_33_MOESM5_ESM.tif]

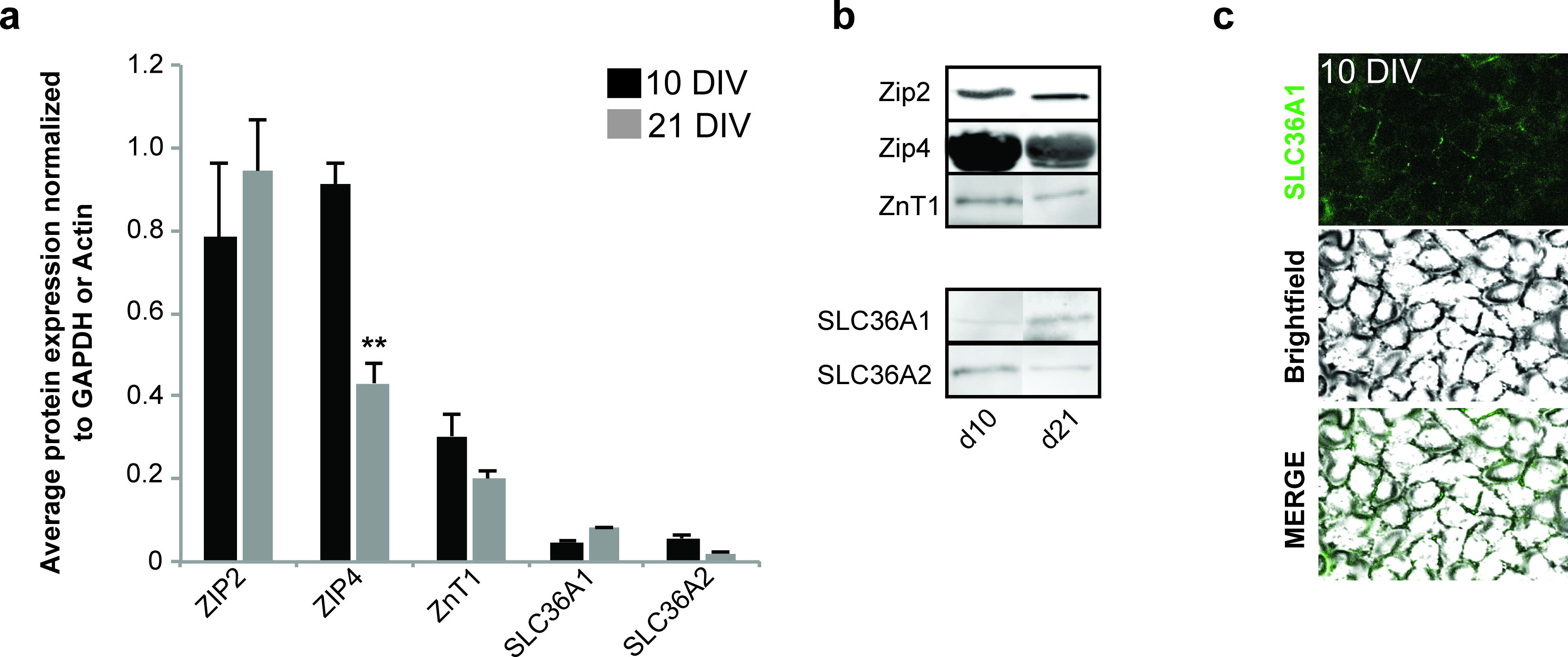

Supplement: Supplementary file 6 — Supplementary material 6 (TIFF 3212 kb) [file 10534_2017_33_MOESM6_ESM.tif]
